# Cyclooxygenase‐2 facilitates dengue virus replication and serves as a potential target for developing antiviral agents

**DOI:** 10.1038/srep44701

**Published:** 2017-03-20

**Authors:** Chun-Kuang Lin, Chin-Kai Tseng, Yu-Hsuan Wu, Chih-Chuang Liaw, Chun-Yu Lin, Chung-Hao Huang, Yen-Hsu Chen, Jin-Ching Lee

**Affiliations:** 1 Doctoral Degree Program in Marine Biotechnology, College of Marine Sciences, National Sun Yat-Sen University, Kaohsiung, Taiwan; 2 Doctoral Degree Program in Marine Biotechnology, Academia Sinica, Taipei, Taiwan; 3 Institute of Basic Medical Sciences, College of Medicine, National Cheng Kung University, Tainan, Taiwan; 4 Center of Infectious Disease and Signaling Research, College of Medicine, National Cheng Kung University, Tainan, Taiwan; 5 Department of Marine Biotechnology and Resources, College of Marine Sciences, National Sun Yat-Sen University, Kaohsiung, Taiwan; 6 School of Medicine, College of Medicine, Kaohsiung Medical University, Kaohsiung, Taiwan; 7 Division of Infectious Diseases, Department of Internal Medicine, Kaohsiung Medical University Hospital, Kaohsiung, Taiwan; 8 School of Medicine, Graduate Institute of Medicine, Sepsis Research Center, Center for Dengue Fever Control and Research, Kaohsiung Medical University, Kaohsiung, Taiwan; 9 Department of Biological Science and Technology, College of Biological Science and Technology, National Chiao Tung University, HsinChu, Taiwan; 10 Department of Biotechnology, College of Life Science, Kaohsiung Medical University, Kaohsiung, Taiwan; 11 Graduate Institute of Natural Products, College of Pharmacy, Kaohsiung Medical University, Kaohsiung, Taiwan; 12 Research Center for Natural Products and Drug Development, Kaohsiung Medical University, Kaohsiung, Taiwan

## Abstract

Cyclooxygenase-2 (COX-2) is one of the important mediators of inflammation in response to viral infection, and it contributes to viral replication, for example, cytomegalovirus or hepatitis C virus replication. The role of COX-2 in dengue virus (DENV) replication remains unclear. In the present study, we observed an increased level of COX-2 in patients with dengue fever compared with healthy donors. Consistent with the clinical data, an elevated level of COX-2 expression was also observed in DENV-infected ICR suckling mice. Using cell-based experiments, we revealed that DENV-2 infection significantly induced COX-2 expression and prostaglandin E_2_ (PGE_2_) production in human hepatoma Huh-7 cells. The exogenous expression of COX-2 or PGE_2_ treatment dose-dependently enhanced DENV-2 replication. In contrast, COX-2 gene silencing and catalytic inhibition sufficiently suppressed DENV-2 replication. In an ICR suckling mouse model, we identified that the COX-2 inhibitor NS398 protected mice from succumbing to life-threatening DENV-2 infection. By using COX-2 promoter-based analysis and specific inhibitors against signaling molecules, we identified that NF-κB and MAPK/JNK are critical factors for DENV-2-induced COX-2 expression and viral replication. Altogether, our results reveal that COX-2 is an important factor for DENV replication and can serve as a potential target for developing therapeutic agents against DENV infection.

Dengue virus (DENV) is a rapidly spreading mosquito-borne viral disease that is dispersed throughout the tropical and subtropical world^1^. At present, there are approximately 400 million DENV-infected patients and 2.5 billion individuals are at risk of DENV infection in the world^2^. DENV infection causes acute human diseases ranging from a self-limiting illness called dengue fever (DF) to a life-threatening form of dengue hemorrhagic fever (DHF) and dengue shock syndrome (DSS)^3^. The therapeutic strategy for DENV-infected patients and the prevention of a second infection by a different serotype of DENV are not only medical problems but also a critical sanitation issue. The ideal DENV vaccine should provide long-term protection against each of the four DENV serotypes to avoid the risk of antibody-dependent enhancement (ADE), and it should induce fewer side effects caused by cross-reactive antibodies^4^. To date, no effective DENV vaccine against all 4 serotypes is available. Therefore, a potential viral or host target for developing anti-DENV agents has become a strong medical needed.

DENV belongs to the *Flavivirus* genus within the *Flaviviridae* family. DENV is a mosquito-borne virus and contains a positive-stranded genome with approximately 11 kilobases (kb) flanked by two structural untranslated regions (UTRs) located at the 5′ and 3′ ends^5^. The DENV RNA genome encodes a single polyprotein that is subsequently cleaved by both host and virus proteases into at least 10 mature individual proteins, including structural proteins (C, prM, and E) and nonstructural proteins (NS1, NS2A, NS2B, NS3, NS4A, NS4B, and NS5)^6^.

COX-2 is a rate-limiting enzyme in the conversion of arachidonic acid to prostaglandins (PGs) G_2_ and H_2_, and PGH_2_ is rapidly converted into more stable PGs. This conversion is being dependent on tissue-specific synthases, including PGD_2_, PGE_2_, PGF_2_, and PGI_2_ (prostacyclins)^7^. Accumulating evidence indicates that PGE_2_ activates downstream cellular mediators via autocrine or paracrine signaling, leading to inflammation-related disease pathogenesis, including metastasis, transformation, and angiogenesis^8^,^9^. Furthermore, the COX-2/PGE_2_ signaling cascade is highly modulated by various viral infections, including hepatitis C virus (HCV)^10^, enterovirus 71 (EV71)^11^, cytomegalovirus (CMV)^12^, and hepatitis B virus (HBV)^13^, contributing to virus replication and viral pathogenesis through elevated COX-2/PGE_2_ expression. Several reports have shown that the suppression of COX-2 expression and PGE_2_ production by selective COX-2 inhibitors or non-steroidal anti-inflammatory drugs (NSAIDs) has an antiviral effect, attenuating disease severity in, for example, herpes simplex virus (HSV)^14^, influenza H5N1^15^, Japanese encephalitis virus (JEV)^16^, EV71^17^, and HCV infections^18^. Recent studies revealed that DENV triggered dendritic cell (DC) migration through regulation of the COX-2-dependent signaling cascade, which might facilitate spreading of DENV to different tissues^19^. The role of COX-2/PGE_2_ in DENV replication remains to be investigated.

To address the correlation between COX-2 and DENV, we first analyzed the status of COX-2 activation upon DENV infection in clinical samples and an animal model. Then, we utilized a reporter-based DENV replicon and a DENV infection system to evaluate the activation of COX-2 during DENV replication. The level of COX-2 and its product PGE_2_ induced by DENV infection were quantified at different time points and viral loads. The effects of exogenous COX-2 expression or added PGE_2_ on DENV replication were determined. Furthermore, COX-2 short hairpin RNA (shRNA) and the specific inhibitor NS398 were used to determine whether COX-2 plays an important role in DENV replication. The detailed regulation of COX-2 activation by DENV infection was investigated. Finally, the effect of a COX-2-specific inhibitor on an ICR suckling mouse model was investigated to evaluate the potency of COX-2 as a therapeutic target against DENV infection.

## Results

### COX-2 levels are elevated in patients with DF

**Figure 1:**
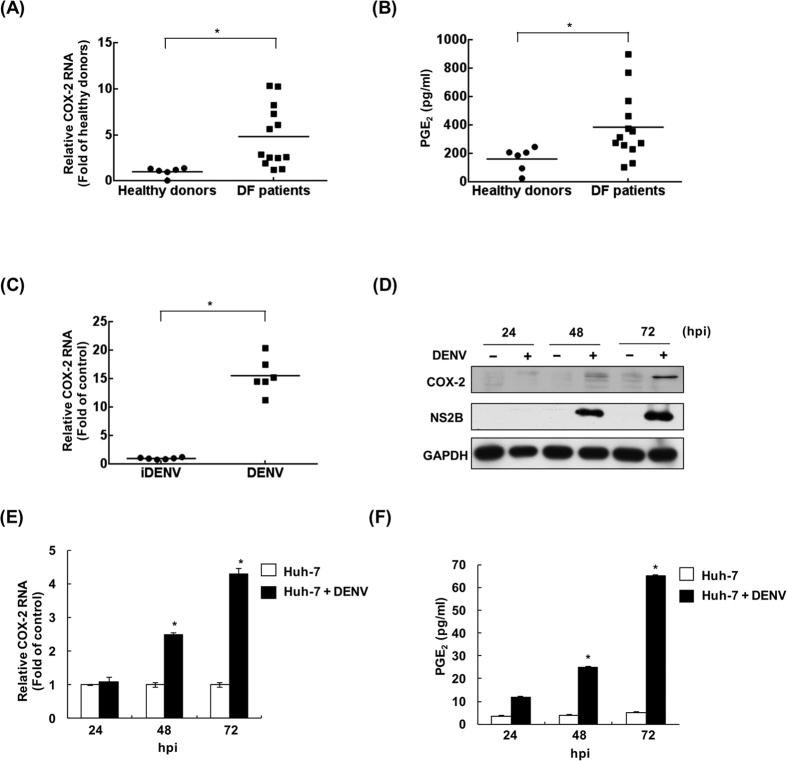
DENV induces COX-2 expression and PGE_2_ production in DF patients, DENV-infected mice, and human hepatoma cells. (**A** and **B**) Elevated COX-2 expression and PGE_2_ levels in the blood of dengue fever patients. COX-2 mRNA and PGE_2_ levels in blood samples from 13 clinical DF patients and 6 healthy donors were determined by RT-qPCR or ELISA, respectively. (**C**) The induced COX-2 expression in DENV-2-infected ICR suckling mice. Six-day-old suckling mice were injected with 2.5 × 10^5^ pfu of DENV-2 or heat-inactivated DENV-2 (iDENV) by intracerebral injection. Each group comprised six suckling mice (n = 6). Six days after inoculation, COX-2 mRNA levels of mouse brain tissues were determined by RT-qPCR. DENV-2 time-dependently induced (**D**) COX-2 protein expression, (**E**) COX-2 RNA replication, and (**F**) PGE_2_ production. Huh-7 cells were infected with DENV-2 at an MOI of 0.1, and the cell lysate and cellular RNA were extracted at the indicated time points (24, 48, and 72 hpi). Western blotting was performed with anti-COX-2, anti-NS2B, and anti-GAPDH antibodies. Relative RNA levels of DENV-2 and COX-2 were determined by RT-qPCR following the normalization of cellular *gapdh* mRNA levels. Supernatants were collected at the indicated time points and subjected to a PGE_2_ ELISA assay. All data from cell-based experiments are indicative of at least three independent experiments, with each measurement carried out in triplicate. Error bars are expressed as the mean ± SD of three independent experiments; *P < 0.05.

To examine the levels of COX-2 gene expression and its metabolite PGE_2_ during DENV infection, blood samples from 13 patients with DF and 6 healthy donors were analyzed by RT-qPCR and ELISA, respectively. Elevated levels of COX-2 RNA and PGE_2_ were observed in 11 out of 13 patients with DF, compared with the group of healthy donors (Fig. 1A and B). These patients were also diagnosed with DHF, and 10 out of 11 patients presented with plasma leakage, which exhibited higher COX-2 and PGE_2_ levels. For 2 out of the 13 patients, these was no significantly induction of COX-2 expression or PGE_2_ production. Taking these observations together, we suggested that the levels of COX-2 and PGE_2_ production might correlate with the severity of the disease.

### DENV-2 infection induces COX-2 expression in ICR suckling mice

To investigate the COX-2 expression level in a DENV-2-infected mouse model, we used DENV-2 or heat-inactivated DENV-2 virus (iDENV) to intracerebrally inject the 6-day-old ICR suckling mice with 2.5 × 10^5^ pfu. Similar to the observations from the clinical samples, the level of COX-2 in brain tissue was significantly increased by DENV-2 infection at 6 days post-infection (dpi), compared with iDENV-infected mice, according to RT-qPCR (Fig. 1C).

### DENV-2 infection induces COX-2 expression and PGE_2_ production in hepatoma cells

To demonstrate COX-2 activation by DENV-2 infection, we first examined the protein and RNA levels of COX-2 in DENV-2-infected Huh-7 cells at different time points. The Results of western blotting and RT-qPCR indicated time-dependent induction of COX-2 protein and RNA levels caused by DENV-2 infection (Fig. 1D and E). Then, the amount of the COX-2-metabolite PGE_2_ was determined by ELISA. As shown in Fig. 1F, PGE_2_ production was also induced in a time-dependent manner. We further confirmed the inductive effect of DENV-2 infection on COX-2 expression by using different concentrations of virus. The results indicated that the levels of COX-2 protein, RNA, and its metabolite PGE_2_ were induced by DENV-2 infection in a concentration-dependent manner (Fig. S1). To further confirm the direct activation of COX-2 by DENV-2 infection, we examined the DENV-2-induced COX-2 expression at 24 hours post-infection (hpi). The results of western blotting and RT-qPCR analysis showed that COX-2 expression can be significantly induced by DENV-2 infection at early time point (Fig. S2A and B).

### COX-2 overexpression and the addition of PGE2 enhance DENV-2 replication

**Figure 2:**
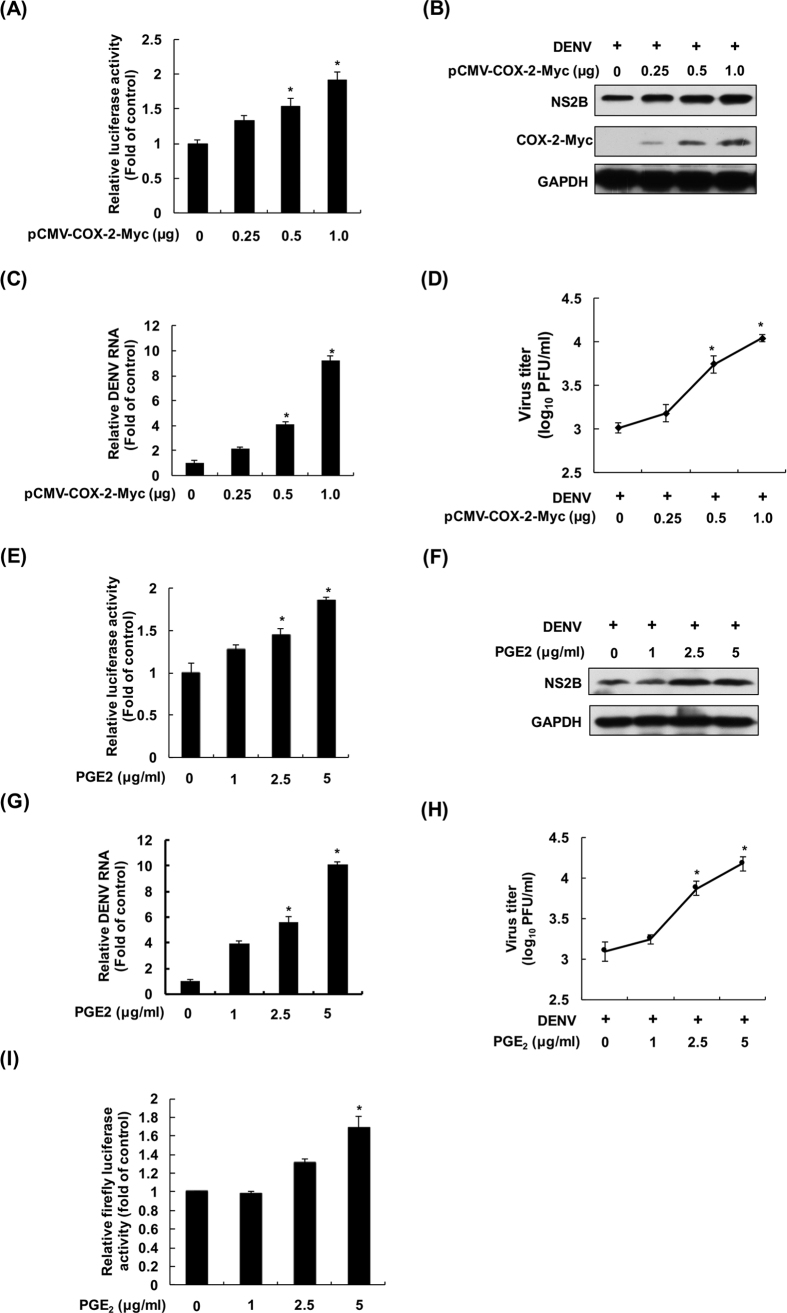
COX-2 overexpression and PGE_2_ treatment increase DENV-2 replication. COX-2 overexpression induced DENV-2 replication in (**A**) DENV-2 replicon cells and (**B** and **C**) the DENV infection system. Huh-7-D2-FLuc-SGR-Neo DENV replicon reporter cells were transfected with pcDNA4/Myc or pcDNA4-COX-2-Myc at the indicated concentrations. After 3 days of incubation, the cell lysates were subjected to a luciferase activity assay. Huh-7 cells were transfected with pcDNA4/Myc or pcDNA4-COX-2-Myc at the indicated concentrations, and the transfected cells were infected with DENV-2 at an MOI of 1. After 3 days of incubation, the cell lysates and cellular RNA were subjected to western blotting and RT-qPCR. (**D**) COX-2 overexpression increased DENV-2 propagation. The transfected Huh-7 cells were infected by DENV-2 at a MOI of 1 for 3 days. Supernatants were collected and subjected to a viral plaque assay. PGE_2_ treatment induced DENV-2 replication in (**E**) viral replicon cells and (**F** and **G**) the DENV infection system. Huh-7-D2-FLuc-SGR-Neo DENV replicon reporter cells were treated with PGE_2_ at the indicated concentrations for 3 days and the cell lysates were subjected to a luciferase activity assay. Huh-7 cells were infected with DENV-2 at an MOI of 1, and the infected cells were treated with PGE_2_ at the indicated concentrations for 3 days. Western blotting was performed with anti-NS2B, anti-Myc, and anti-GAPDH antibodies. Relative RNA levels of DENV-2 was determined by RT-qPCR following the normalization of cellular *gapdh* mRNA levels. (**H**) PGE_2_ treatment induced DENV-2 propagation. Huh-7 cells were infected with DENV-2 at an MOI of 1 and then treated with PGE_2_. Supernatants were collected and subjected to a viral plaque assay. (**I**) PGE_2_ treatment induced DENV-2 NS5 polymerase activity. Huh-7 cells were cotransfected with p(+)RLuc-(−)DV-UTRΔC-Fluc reporter template (0.5 μg) and pcDNA-NS5-Myc expression plasmid (0.5 μg), and the transfected cells were treated with PGE_2_ at the indicated concentrations for 3 days. The cell lysates were subjected to a Dual-Glo Luciferase Assay. All data were indicative of at least three independent experiments, with each measurement carried out in triplicate. Error bars are expressed as the mean ± SD of three independent experiments; *P < 0.05.

To evaluate whether COX-2 induction facilitates DENV-2 replication, we first transfected Huh-7-D2-FLuc-SGR-Neo DENV replicon reporter cells with either vehicle vector pcDNA4/Myc or COX-2 expression vector pCMV-COX-2-Myc. After 3 days of incubation, cell lysates were subjected to a luciferase activity assay. As shown in Fig. 2A, overexpression of COX-2 concentration-dependently enhanced luciferase activity, indicating that DENV-2 replication was elevated by exogenous COX-2 expression. Then, the DENV infection system was used to confirm the effect of exogenous COX-2 expression on DENV-2 replication. We first determined the growth curve of the DENV-2 infection system (Fig. S3A). Then, the vehicle vector- or pCMV-COX-2-Myc-transfected Huh-7 cells were infected with DENV-2. After 3 dpi, the protein level of DENV-2 was also elevated with an increased level of exogenous COX-2 expression (Fig. 2B). Similarly, the level of viral RNA replication was elevated 9.6-fold in the pCMV-COX-2-Myc-transfected cells compared with the vehicle-transfected cells (Fig. 2C). The virus loads in the supernatants of vehicle- or pCMV-COX-2-Myc-transfected Huh-7 cells were determined with a plaque assay, and the results showed that exogenous COX-2 expression enhanced viral propagation approximately 1.8- to 10-fold in a concentration-dependent manner (Fig. 2D). In addition, we also examined the viral load in the supernatants of vehicle- or pCMV-COX-2-Myc-transfected Huh-7 cells at an early time point, and the results showed that COX-2 concentration-dependently elevated DENV-2 propagation at 24 hpi. (Fig. S3B). To investigate whether the COX-2-metabolite PGE_2_ has an impact on DENV-2 replication, Huh-7-D2-FLuc-SGR-Neo replicon reporter cells were incubated with PGE_2_ at the indicated concentrations for 3 days. As shown in Fig. 2E, luciferase activities were enhanced with increasing concentrations of PGE_2_, indicating that PGE_2_ could elevate DENV replication. Subsequently, we utilized the DENV infection system to confirm the inductive effect of PGE_2_ on DENV-2 replication. Consistently, PGE_2_ dose-dependently elevated the DENV-2 protein level in DENV-2-infected Huh-7 cells (Fig. 2F). The results of RT-qPCR showed that PGE_2_ dose-dependently induced DENV-2 RNA replication, with a 10.3-fold change in the viral RNA level at 5 μg/ml of PGE_2_ treatment compared with the PGE2-untreated cells (Fig. 2G). The virus loads in the supernatants of PGE_2_-treated Huh-7 cells were determined with a plaque assay, and the results showed that the viral titer of the PGE_2_-treated cells at 5 μg/ml was approximated 12-fold higher than that of the PGE_2_-untreated cells (Fig. 2H). In addition, we also determined the viral load at an early time point in PGE_2_-treated cells, and the result showed that PGE_2_ increased DENV-2 propagation with approximately a log elevation change (Fig. S3C). To evaluate the possible mechanism of the PGE_2_-increased DENV-2 replication, we examined the effect of PGE_2_ on DENV-2 NS2B/NS3 protease and NS5 polymerase activity. Both viral proteins are critically responsible for viral replication. In the reporter assay for DENV protease, the increasing concentrations of PGE_2_ had no effect on protease activity (Fig. S4). In the reporter assay for DENV-2 polymerase, Huh-7 cells were cotransfected with the p(+)RLuc-(−)DV-UTRΔC-Fluc reporter template and the DENV-2 NS5 expression vector, followed by incubation with PGE_2_ for 3 days. As shown in Fig. 2I, NS5-mediated RLuc synthesis was slightly induced by PGE_2_, with a 1.7-fold change at 5 μg/ml of PGE_2_ treatment compared with PGE_2_-untreated cells. These results might indicate that the COX-2 metabolite PGE_2_, in promoting DENV-2 polymerase activity, partially participate in DENV-2 replication.

### DENV-2-elevated COX-2 expression and PGE2 production are required for DENV-2 replication

**Figure 3:**
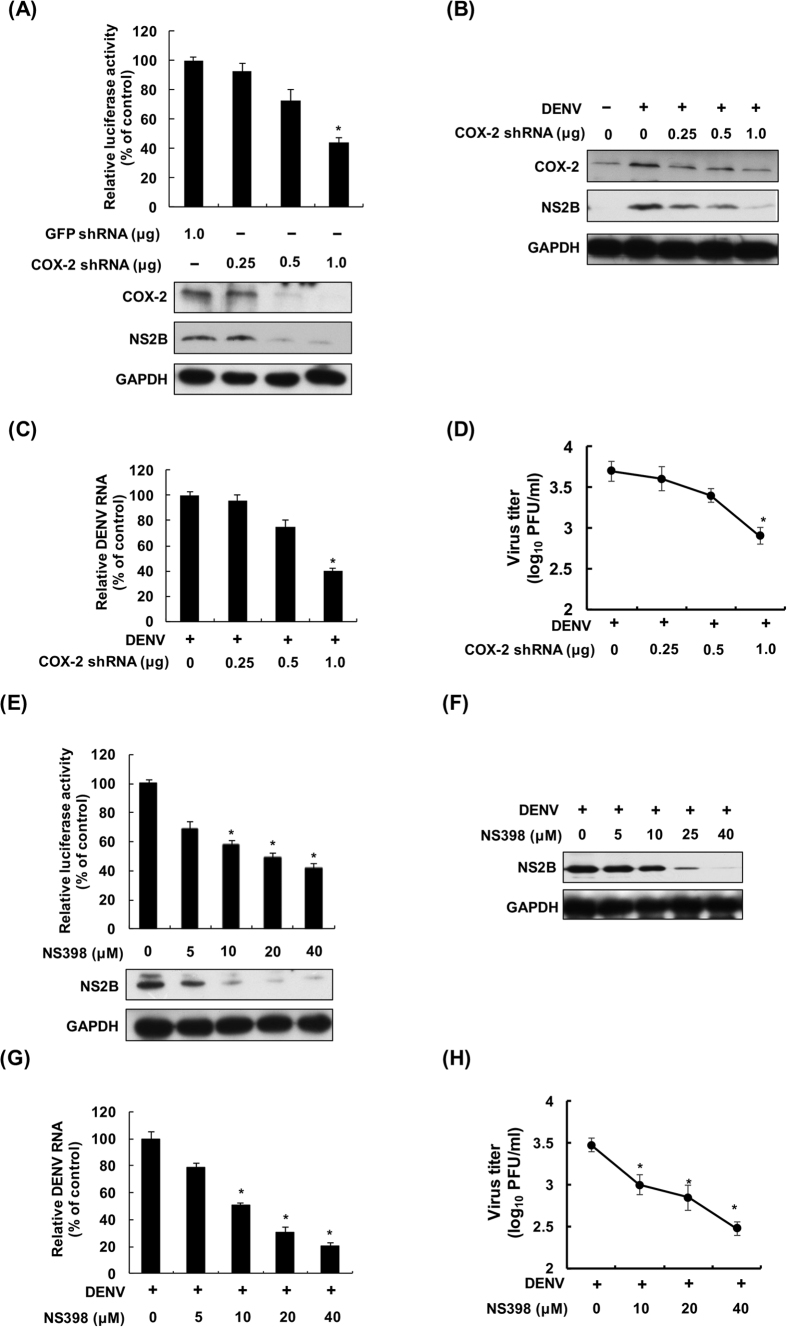
Reduction of COX-2 expression and catalytic activity by shRNA or NS398 reduce DENV replication. COX-2 shRNA reduced DENV replication in (**A**) DENV replicon cells and (**B** to **D**) a DENV infection system. Huh-7-D2-FLuc-SGR-Neo DENV replicon cells were transfected with GFP or COX-2 shRNA at the indicated concentrations for 3 days and the cell lysates were subjected to a luciferase activity assay and western blotting. Huh-7 cells were transfected with COX-2 shRNA at the indicated concentrations, and the transfected cells were infected with DENV-2 at an MOI of 1. After 3 days of treatment, the cell lysate, cellular RNA and supernatants were analyzed by western blotting, RT-qPCR or plaque assay, respectively. NS398 reduced DENV replication in (**E**) DENV replicon cells and (**F** to **H**) a DENV infection system. Huh-7-D2-FLuc-SGR-Neo DENV replicon cells were treated with NS398 at different concentrations (0, 5, 10, 20, and 40 μM) for 3 days, and the cell lysates were subjected to a luciferase activity assay and western blotting. Huh-7 cells were infected with DENV-2 at an MOI of 1 and then treated with NS398 at different concentrations (0, 5, 10, 20, and 40 μM) for 3 days. Western blotting was performed with anti-COX-2, anti-NS2B, and anti-GAPDH antibodies. The relative RNA level of DENV-2 was determined by RT-qPCR following normalization to the cellular *gapdh* mRNA level. All data are indicative of at least three independent experiments, with each measurement performed in triplicate. Error bars are expressed as the mean ± SD of three independent experiments; *P < 0.05.

To clarify the role of COX-2 in DENV-2 replication, we examined the effect of COX-2 silencing in DENV-2 replication using Huh-7-D2-FLuc-SGR-Neo DENV replicon reporter cells. The results of luciferase activity assay and western blotting showed that the COX-2 shRNA attenuated DENV-2 replication and protein synthesis in a concentration-dependent manner (Fig. 3A). Subsequently, we utilized the DENV infection system to confirm the reductive effect of COX-2 shRNA on DENV-2 replication. Consistently, the results of western blotting showed that COX-2 shRNA attenuated DENV-2 replication in a concentration-dependent manner (Fig. 3B). The results of RT-qPCR and a plaque assay showed that COX-2 shRNA reduced DENV-2 RNA replication and propagation in a concentration-dependent manner, with an approximately 60% reductive effect by transfection of 1 μg of the COX-2 shRNA expression vector (Fig. 3C and D). In addition to the genetic silencing approach to investigate the effect of COX-2 expression on DENV-2 replication, we used the enzymatic inhibitor NS398 against COX-2 activity to investigate the important role of COX-2 in DENV-2 replication. We first examined the anti-viral effect of NS398 on DENV-2 entry and assembly. The results revealed that there was no significant reduction in DENV-2 entry and assembly in response to NS398 treatment (Fig. S5A and B). Then, the effect of NS398 on DENV-2 replication was determined in the NS398-treated Huh-7-D2-FLuc-SGR-Neo DENV replicon reporter cells. The results of luciferase activity and western blotting showed that NS398 dose-dependently reduced luciferase activity and DENV-2 protein synthesis (Fig. 3E). To confirm the reductive effect of NS398 in DENV-2 replication, we used the DENV infection system to examine DENV replication in the NS398-treated or untreated DENV-2-infected cells. As shown in Fig. 3F and S6B, NS398 reduced DENV-2 protein synthesis and DENV-2-induced PGE_2_ production in a dose-dependent manner without significant cell cytotoxicity (Fig. S6A). Similarly, NS398 dose-dependently suppressed DENV-2 RNA replication and viral propagation with an approximately 80% inhibitory effect at 40 μM (Fig. 3G and H). To verify whether NS398 affected the viral protein expression, we determined the amount of DENV positive-sense RNA and intracellular NS1 protein in response to NS398 treatment. We calculated the relative inhibitory ratio of viral NS1 protein to positive-sense RNA, and the result revealed that the similar inhibitory effect of NS398 on viral RNA and protein levels (Fig. S6C). Therefore, we suggested that the antiviral effect of NS398 majorly targeted DENV RNA replication machinery, and then leading to decrease in viral protein expression. We further demonstrated that NS398 suppressed DENV-2 propagation at 24 hpi (Fig. S6D). In addition, we demonstrated that NS398 exhibited an inhibitory effect on the replication of four DENV serotypes (Fig. S7).

**Figure 4:**
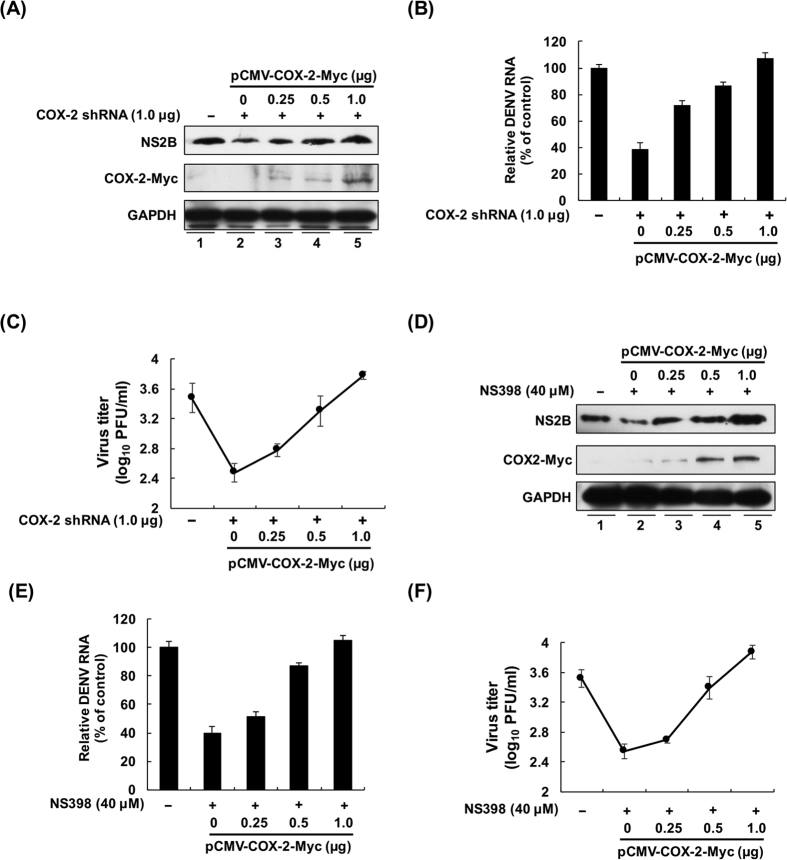
COX-2 expression is required for viral replication. (**A** to **C**) Exogenous COX-2 expression restored DENV-2 protein synthesis, RNA replication and viral propagation in COX-2 shRNA-transfected cells. Huh-7 cells were cotransfected with COX-2 shRNA (1.0 μg) and pCMV-COX-2-Myc (0.25, 0.5, and 1.0 μg), followed by DENV-2 infection at an MOI of 1. (**D** to **F**) Exogenous COX-2 expression restored DENV-2 protein synthesis, RNA replication and viral propagation in NS398-treated cells. Huh-7 cells were transfected with pcDNA4/Myc (0.5 μg) or pCMV-COX-2-Myc (0.25, 0.5, and 1.0 μg), followed by DENV-2 infection at an MOI of 1. The cells were treated with DMSO or 40 μM NS398 for 3 days. Western blotting was performed with anti-NS2B, anti-Myc, and anti-GAPDH antibodies. The relative RNA level of DENV-2 was determined by RT-qPCR following normalization to the cellular *gapdh* mRNA level. All data are indicative of at least three independent experiments, with each measurement performed in triplicate. Error bars are expressed as the mean ± SD of three independent experiments; *P < 0.05.

To further clarify the precise role of COX-2 in DENV-2 replication, we performed a restoration experiment on DENV-2 replication by inducing exogenous COX-2 expression in the presence of COX-2 shRNA expression or the specific inhibitor NS398. In the RNA silencing experiment, GFP shRNA combined with vehicle vector pcDNA4/Myc, as a control, or COX-2 shRNA combined with pCMV-COX-2-Myc were transfected into Huh-7 cells at increasing concentrations (0–1.0 μg/well). Then, all transfected cells were infected with DENV-2. As shown in Fig. 4A, the increasing exogenous COX-2-Myc expression (middle panel, lanes 3–5) gradually restored the DENV-2 protein level inhibited by COX-2 shRNA, compared with the GFP shRNA-transfected cells (lane 1) and COX-2 shRNA-transfected cells (lane 2). Consistently, exogenous COX-2 expression also efficiently restored the COX-2 shRNA-reduced DENV-2 RNA level and viral propagation (Fig. 4B and C). We subsequently examined the effect of COX-2 enzymatic activity on DENV-2 replication using a selective inhibitor. In the enzymatic suppression experiment, pCMV-COX-Myc-transfected cells were treated with or without the COX-2 inhibitor NS398 in the presence of DENV-2 infection. The results of western blotting showed that increasing the amount of COX-2-Myc expression (Fig. 4D, middle panel, lanes 3–5) gradually recovered the inhibitory effect of NS398 on DENV-2 replication (upper panel, lanes 3–5), compared with vehicle vector-transfected cells in the absence or presence of NS398 (lanes 1 and 2). As expected, the greater amount of exogenous COX-2 expression could overcome the inhibitory effect of NS398 on the DENV-2 RNA level and viral propagation (Fig. 4E and F).

### NS398 delays lethality from life-threatening DENV-2 infection in ICR suckling mice

**Figure 5:**
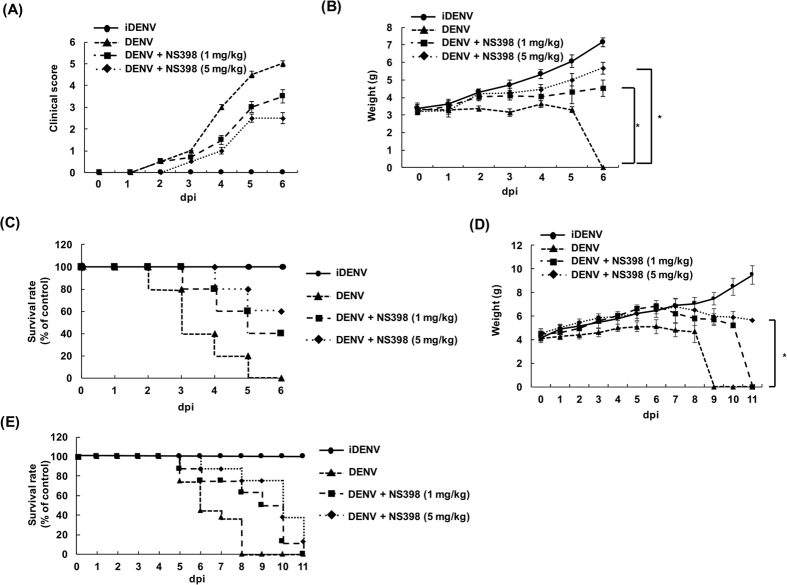
NS398 protects ICR suckling mice from life-threatening DENV-2 infection. Six-day-old suckling mice were injected with 2.5 × 10^5^ pfu of DENV-2 or heat-inactivated DENV-2 (iDENV), as a negative control, by intracerebral injection. Then, NS398 was injected into DENV-2-infected mice at a dose of 1 and 5 mg/kg by intracerebral injection at 1, 3, and 5 dpi. Following inoculation, (**A**) clinical score, (**B**) mouse body weights, and (**C**) survival rate were recorded daily. The symptoms of the clinical score are shown as follows: 0 for no illness symptoms, 1 for ruffled fur and anorexia, 3 for paralysis, 4 for lethargy, and 5 for moribund. (**D**) Body weight and (**E**) survival rate of the DENV-2-infected mice were recorded daily until 11 dpi. Each group comprised 12 suckling mice (n = 12). Error bars are expressed as the mean ± SD of three independent experiments; *P < 0.05.

To determine the therapeutic potential of COX-2 inhibition against DENV *in vivo*, we intracerebrally injected DENV-2 or iDENV into 6-day-old ICR suckling mice with 2.5 × 10^5^ pfu and then inoculated with 1 mg/kg or 5 mg/kg of NS398 at 1, 3, and 5 dpi. The clinical score, mouse body weight, ands survival rate of DENV-2-injected mice with or without NS398 treatment were measured daily for 6 days. As shown in Fig. 5A, the clinical score revealed that DENV-2-infected mice without NS398 treatment continually showed ruffled fur, anorexia, severe paralysis, and lethargy and were moribund within 3–6 dpi, compared with the iDENV-infected mice. Nevertheless, the DENV-2-infected mice with NS398 treatment displayed less severe symptoms within 6 days of inoculation, compared with the DENV-2-infected mice without NS398 treatment. Furthermore, we monitored the weight of the mice daily throughout the 6-day experiment. As shown in Fig. 5B, in DENV-2-infected mice, NS398 treatment consistently reduced the loss of body weight caused by viral infection when compared with the DENV-2-infected mice without NS398 treatment. Notably, NS398 maintained the survival rate of DENV-2-infected mice at 40% and 60% at doses of 1 mg/kg and 5 mg/kg, respectively, at 6 dpi (Fig. 5C). Furthermore, we observed that NS398 treatment expanded the life-span of ICR mice with life-threatening DENV-2 infection by more than 7 days, compared with DENV-infected ICR mice without NS398 treatment following a longer period (Fig. 5D and E). Taken together, the suppression of COX-2 function prolongs life-span and delays DENV-2-induced lethality.

### DENV-2 elevates COX-2 promoter activation through mediation of NF-κB and C/EBP binding elements

**Figure 6:**
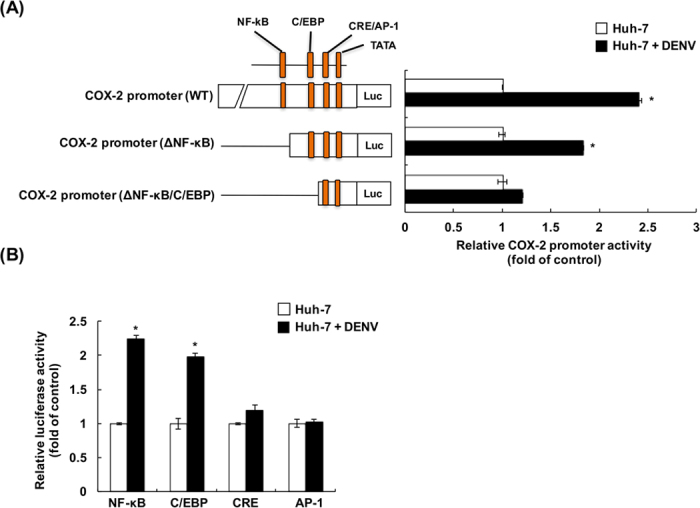
DENV-2 elevates COX-2 promoter activation through mediation of NF-κB and C/EBP binding elements. (**A**) NF-κB and C/EBP were critical binding elements for DENV-2-induced COX-2 promoter activity. Huh-7 cells were cotransfected with 0.2 μg of pCMV-Renilla-Luc and COX-2 promoter-linked firefly luciferase reporter vectors containing various deletions of transcriptional factor binding elements, including WT, ΔNF-κB, and ΔNF-κB/C/EBP. The transfected cells were infected by DENV-2 at an MOI of 1, and the cell lysates were subjected to a Dual-Glo Luciferase Assay at 2 dpi. (**B**) DENV-2 infection induced the activation of NF-κB and C/EBP binding elements. Huh-7 cells were cotransfected with 0.2 μg of pCMV-Renilla-Luc and pNF-κB-Luc, pC/EBP-Luc, pCRE-Luc, or pAP-1-Luc. Then, the transfected cells were infected with DENV-2 at an MOI of 1. The cell lysates were subjected to a Dual-Glo Luciferase Assay at 2 dpi. All data are indicative of at least three independent experiments, with each measurement carried out in triplicate. Error bars are expressed as mean ± SD of three independent experiments; *P < 0.05.

COX-2 induction is regulated by several major transcriptional factors in response to inflammation, such as NF-κB, C/EBP, CRE, and AP-1^20^. To identify the regulatory mechanism of DENV-2-induced COX-2 expression, several COX-2 promoter-linked firefly luciferase reporter vectors containing various deletions of transcriptional factor binding elements, including ΔNF-κB, and ΔNF-κB/C/EBP, were used to investigate the transcriptional factors involved in COX-2 promoter activation. The full-length COX-2 promoter, termed the WT, served as a positive control. As shown in Fig. 6A, a significant induction of luciferase activity was observed in NF-κB, and C/EBP binding element-linked reporters in the presence of DENV-2 infection. In contrast, no significant induction of luciferase activity occurred with the reporter construct containing only the CRE/AP1 binding element. These results indicated that the regions of NF-κB and C/EBP were important regulatory sequences responsible for the activation of the COX-2 promoter by DENV-2 infection. To further separately determine whether NF-κB, C/EBP, CRE, and AP-1 were directly affected by DENV-2 infection, the individual transcription factor binding sites were linked to the luciferase gene to generate reporter constructs. Each reporter construct was transfected into Huh-7 cells, followed by DENV-2 infection. As shown in Fig. 6B, the activities of luciferase linked to either the NF-κB or C/EBP binding region were highly induced by DENV-2 infection; in contrast, the activities of luciferase linked to either the CRE or AP-1 binding region could not be significantly induced by DENV-2 infection.

### NF-κB and MAPK/JNK-mediated C/EBP are responsible for DENV-2-induced COX-2 expression and viral replication

**Figure 7:**
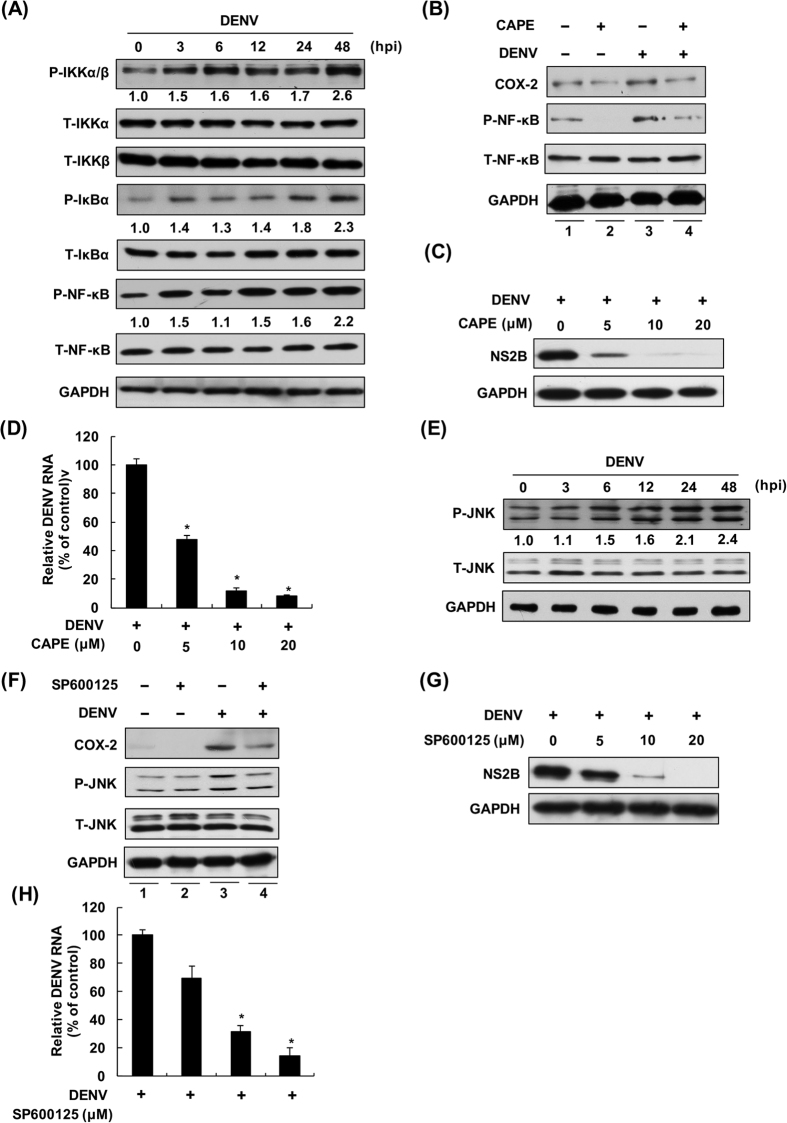
NF-κB and MAPK/JNK-mediated C/EBP are responsible for DENV-2-induced COX-2 expression and viral replication. (**A**) DENV-2 induced activation of the NF-κB signaling pathway. Huh-7 cells were infected by DENV-2 at an MOI of 1 and the cell lysates were extracted at the indicated time points. Western blotting was performed and the relative blot intensities were quantified by densitometry scanning. (**B**) CAPE significantly suppressed DENV-2-induced COX-2 expression. Huh-7 cells were pretreated with DMSO or CAPE (20 μM) for 2 h and then infected with DENV-2 at an MOI of 1. The cell lysates were analyzed at 2 dpi by western blotting. (**C** and **D**) CAPE dose-dependently suppressed DENV-2 protein synthesis and RNA replication. Huh-7 cells were infected by DENV-2 and treated with CAPE at different concentrations (0, 5, 10, and 20 μM). After 3 days of treatment, the cell lysates and cellular RNA were analyzed by western blotting or RT-qPCR, respectively. (**E**) DENV-2 induced the activation of the MAPK/JNK pathway. Huh-7 cells were infected by DENV-2, and the cell lysates were extracted at the indicated time points. Western blotting was performed and the relative blot intensities were quantified by densitometry scanning. (**F**) SP600125 significantly suppressed DENV-2-induced COX-2 expression. Huh-7 cells were pretreated with DMSO or SP600125 (20 μM) for 2 h, and then, the cells were infected by DENV-2. After 2 days of treatment, cell lysates were subjected to western blotting. (**G** and **H**) SP600125 dose-dependently suppressed DENV-2 protein synthesis and RNA replication. Huh-7 cells were infected by DENV-2, and the cells were treated with DMSO or SP600125 at different concentrations (0, 5, 10, and 20 μM). After 3 days of treatment, the cell lysates and cellular RNA were analyzed by western blotting or RT-qPCR, respectively. GAPDH served as a loading control in western blotting. The relative RNA level of DENV-2 was determine by RT-qPCR following normalization to the cellular *gapdh* mRNA level. All data are indicative of at least three independent experiments, with each measurement carried out in triplicate. Error bars are expressed as the mean ± SD of three independent experiments; *P < 0.05.

To investigate the details of NF-κB signaling pathway involvement in DENV-2-induced COX-2 activation, we first examined the phosphorylation level of the IKK complex, IκB, and NF-κB in a time course analysis using western blotting. As shown in Fig. 7A, DENV-2 significantly increased the phosphorylation level of the IKK complex (upper panel), IκB (middle panel), and NF-κB (lower panel) at 3, 12, 24, and 48 hpi, revealing that a greater amount of activated NF-κB can translocate into the nucleus to drive COX-2 expression because of proteasome degradation of phospho-IκBα. To confirm that DENV-2-activated NF-κB is involved in COX-2 expression, CAPE, an inhibitor of NF-κB activation, was used to analyze the role of NF-κB in DENV-2-induced COX-2 expression. Huh-7 cells were pretreated with DMSO or CAPE, followed by DENV-2 infection. As shown in Fig. 7B, CAPE reduced DENV-2-induced COX-2 expression (lanes 3 and 4). To further evaluate the effect of CAPE on DENV-2 replication, DENV-2-infected cells were treated with CAPE at different concentrations. After 3 days of treatment, total cell lysates and cellular RNA were collected and subjected to western blotting and qRT-PCR. The results showed that CAPE dose-dependently suppressed DENV-2 protein synthesis (Fig. 7C) and RNA levels (Fig. 7D), respectively. Therefore, we concluded that activation of the NF-κB signaling pathway is involved in DENV-2-induced COX-2 expression and viral replication.

In addition to the NF-κB signaling pathway, COX-2 activation is mediated by the MAPK/c-Jun N-terminal kinase (JNK) signaling pathway. We first examined whether the phosphorylation level of MAPK/JNK was increased during DENV-2 infection. The cell lysates of DENV-2-infected Huh-7 cells were collected at specific time points. The results of western blotting showed that DENV-2 significantly increased the phosphorylation level of MAPK/JNK at 6, 12, 24, and 48 hpi (Fig. 7E). To further investigate whether the activation of MAPK/JNK was involved in COX-2 expression by activating C/EBP expression during DENV-2 infection, the effect of the JNK- specific inhibitor SP600125 on DENV-2-induced COX-2 expression was analyzed by western blotting. The SP600125-pretreated Huh-7 cells were then infected with DENV-2. As shown in Fig. 7F, SP600125 significantly reduced DENV-2-induced COX-2 expression (lanes 3 and 4). The effect of SP600125 on DENV-2 replication was examined in DENV-2-infected Huh-7 cells upon treatment with different concentrations of SP600125. The total cellular lysates and RNA were subjected to western blotting and qRT-PCR at 3 dpi. The results showed that SP600125 decreased DENV-2 protein (Fig. 7G) and RNA levels (Fig. 7H) in a dose-dependent manner. MAPK/extracellular signal-regulated kinase (ERK) and p38 are two other MAPK signaling pathways involved in regulating COX-2 expression. In the present study, we found that DENV-2 could elevate the phosphorylation level of MAPK/ERK and p38 from 3 to 48 hpi (Fig. S8A). However, the p38 inhibitor SB203580 and ERK inhibitor PD98059 did not reduce DENV-2-induced COX-2 expression (Fig. S8B and C; lanes 3 and 4). Our results were contrasted with the previous results published by Wu *et al*. They reported that SB203580 and PD98059 could effectively reduce the expression of COX-2 in DENV-infected dendritic cells (DCs). In fact, we performed our experiments under the same conditions described by Wu *et al*. following DENV infection at an MOI of 5 and treatment with protein-specific inhibitors for 24 h. The results of western blotting showed that DENV-2 replication and DENV-2-elevated COX-2 expression was reduced by SP600125 and CAPE, but not by SB203580 or PD98059 (Fig. S11), which is consistent with our findings in the compound-treated Huh-7 cells at 72 hpi described above. Therefore, the opposing results might be due to the different cell line used in each experiment. Furthermore, neither inhibitor exhibited no significant suppression of DENV-2 protein synthesis or RNA replication (Fig. S9). Taken together, these results indicate that MAPK/JNK, not ERK or P38, is a critical pathway responsible for DENV-2-induced COX-2 expression and viral replication.

## Discussion

In inflammatory responses caused by viral infection, the COX-2 metabolite PGE_2_ has been reported to be highly associated with viral replication and virulence^21^. In the present study, we observed that COX-2 expression was increased in DENV-infected patients and ICR suckling mice, an observation that was confirmed by cell-based assays using human hepatoma cells (Fig. 1). In addition, COX-2 induction could facilitate DENV-2 propagation by using exogenous overexpression of COX-2 or PGE_2_ treatment, in which PGE_2_-induced DENV-2 polymerase activity might be one of the mechanisms associated with DENV-2 replication (Fig. 2). With the evidence of COX-2 elevation by DENV-2 infection at an early time point (Fig. S2A and B), we suggested that COX-2 can directly affect DENV-2 replication. However, previous studies showed that DENV-mediated elevation of PGE_2_ had no significant influence on DENV propagation in A549 cells, a human lung adenocarcinoma cell line^22^. These different results might be due to different effects of PGE_2_ on DENV replication in different cell types. Therefore, the impact of PGE_2_ on DENV propagation will be further investigated using primary human monocyte cells. In addition to the enhancement of DENV polymerase activity, PGE_2_ was reported to promote EV71 and HIV-1 replication by increasing the level of cyclic adenosine 3′, 5′-monophosphate (cAMP)^23^. The impact of PGE_2_-mediated cAMP levels on DENV replication or DENV polymerase activity is an alternative important issue to be further investigated.

At present, many direct-acting antivirals (DAAs) against viral protein achieve a good sustained virologic response (SVR) in clinical treatment, such as in HCV and HIV treatment^24^,^25^. However, the rapid occurrence of viral resistance is another emergent problem of these DAAs due to the high mutation rate of the viruses caused by the higher replication rate and an error-prone RNA polymerase^26^. Single point mutations in viral proteins can be fully adequate to bypass antiviral agents with high-affinity binding^27^. Therefore, targeting of the host factors required for viral replication is currently considered a promising strategy for drug development, one that overcomes the drug resistance problem due to the low mutational rate within eukaryotic cells^28^,^29^. Furthermore, these host-targeting agents can provide broader genotypic coverage to all virus genotypes or serotypes^30^. Our study first verified that COX-2 is a host factor critical for DENV-2 replication (Figs 3 and 4) and showed that COX-2-mediated DENV replication can be effectively suppressed by the COX-2-specific inhibitor NS398 *in vivo* (Fig. 5). Furthermore, we also demonstrated that DENV-2 replication can be suppressed by NS398 in human monocyte cell line, including THP-1 and U937 cells (Fig. S10). Therefore, our findings provide an attractive host target for developing therapeutic inhibitors against DENV infection.

The risk of hemorrhage and plasma leakage is interrelated with the cross-reactive, non-neutralizing antibodies that present upon heterotypic second infection, resulting in increased deleterious inflammation, viral load, and endothelial dysfunction^31^,^32^. Several inflammatory cytokines, including tumor necrosis factor alpha (TNF-α), interferon-γ, and IL-6, are reported to be highly released in patients with DHF, which is a clinical characteristic of DHF^33^,^34^. Although aberrant activation of COX-2 is associated with severe inflammation and viral pathogenesis, the role of COX-2 in the pathogenesis of DHF remains unclear. Yu-Ping Zhang *et al*. previously observed a high level of COX-2 at the site of hemorrhagic injury^35^. Other research groups demonstrated that the inhibition of COX-2 expression and PGE_2_ production could alleviate hemorrhagic gastrointestinal^36^, brain^37^, and bladder-related injury to improve survival *in vivo*^38^. In the present study, we also observed high elevation of COX-2 expression and its metabolite PEG_2_ in a DF patient (Fig. 1) and demonstrated a reduction in DENV replication and mortality rate by suppression of COX-2 in DENV-infected mice (Figs 3, 4 and 5). It will be interesting to further investigate whether blocking COX-2 can attenuate vascular leakage using a DENV-infected AG129 mouse model for hemorrhagic studies.

**Figure 8:**
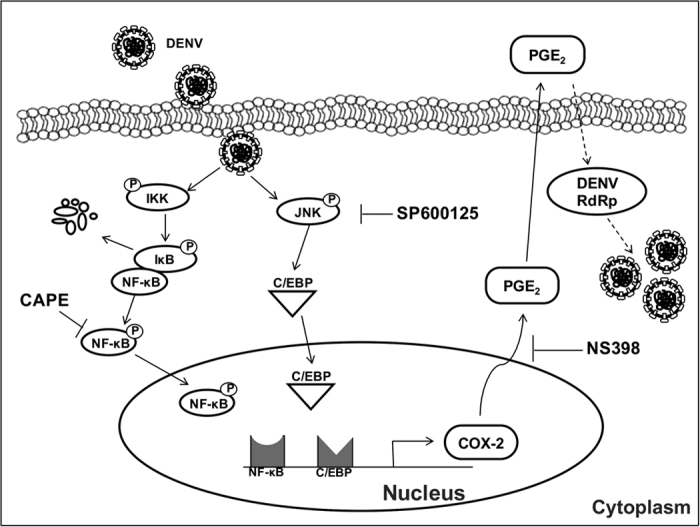
Proposed model to illustrate the mechanism of increased COX-2 expression and PGE_2_ production during DENV infection. DENV infection induces COX-2 expression through activation of the NF-κB and MAPK/JNK-mediated C/EBP signaling pathways. Phosphorylated NF-κB and activated C/EBP are translocated into the nucleus and bind to the COX-2 promoter region. COX-2 induction and elevated PGE_2_ production by DENV infection lead to enhanced activity of the viral polymerase and viral propagation.

In conclusion, our data illustrate a model of DENV-mediated COX-2 expression that facilitates virus propagation (Fig. 8) and provide a potential strategy for the development of a therapeutic agent against DENV replication and inflammation by targeting the expression or catalytic activity of the host factor COX-2.

## Materials and Methods

### Ethics statement

Breeder mice of the ICR strain were obtained from BioLasco Taiwan Co. Ltd, and 6-day-old suckling mice were used in the present study. All animal experiments were performed according to the Guide for the Care and Use of Laboratory Animals. The experimental protocol was approved by the Animal Research committee of Kaohsiung Medical University of Taiwan (IACUC #104032) under the guidance of the Public Health Service (PHS) policy on Human Care and Use of Laboratory Animals. The animals were cared for and raised under standard laboratory conditions according to the Animal Use Protocol of Kaohsiung Medical University and the guidelines established by the Ministry of Science and Technology, Taiwan. Laboratory-confirmed adult dengue patients were prospectively enrolled in Kaohsiung Medical University Hospital from July 2014 to January 2016. All patients in the group had DF and all methods used in the human study according to the criteria defined by the 2009 WHO guidelines^39^ and Centers for Disease Control, Department of Health (Taiwan). Patients’ personal information, clinical data, and medical records were collected, and the study was approved by the Institutional Review Board of Kaohsiung Medical University Hospital (IRB Number: KMUH-IRB-20110451). Informed consent was obtained from all subjects.

### COX-2 expression and PEG2 level in patients with DF

A laboratory-confirmed dengue patient was a patient who had at least two positive results from four experimental examinations, including a real-time PCR assay, dengue NS1 Ag STRIP test, capture IgM and IgG ELISA assay, and dengue viral culture assay. Blood samples were obtained at the febrile phase (from the day symptoms presented to the day 1st after symptoms occurred) and were used to analyze COX-2 expression and PGE_2_ production. All samples were maintained at −80 °C. Serum samples from healthy donors were used to compare COX-2 expression and PGE_2_ production.

### Cell culture and virus

Human monocytic THP-1 cells were maintained in RPMI medium 1640 (RPMI; Invitrogen Life Technologies, Rockville, MD) with L-glutamine and supplemented with 10% fetal bovine serum and 1% antibiotic-antimycotic. Naïve Huh-7 cells and the DENV replicon cell line (Huh-7-D2-FLuc-SGR-Neo)^40^, Huh-7 cells harboring the DENV serotype 2 subgenome (D2-FLuc-SGR-Neo), were cultured in Dulbecco’s modified Eagle’s medium (DMEM; Invitrogen Life Technologies, Rockville, MD) supplemented with 10% fetal bovine serum, 1% non-essential amino acids and 1% antibiotic-antimycotic, and incubated at 37 °C with 5% CO_2_. Different serotype of DENV (DENV-1: DN8700828; DENV-2: DN454009A; DENV-3: DN8700829A; DENV-4: S9201818) were obtained from the Centers of Disease Control, Department of Health, Taiwan. DENV was amplified in C6/36 cells^41^.

### Reagents

NS398^42^, SP600125 (JNK inhibitor II, JNK inhibitor), and caffeic acid phenethyl ester (CAPE, NF-κB inhibitor)^43^ were purchased from Sigma Chemical Co (St. Louis, MO, USA). SB203580 (p38 inhibitor) and PD98059 (ERK inhibitor) were obtained from Cell Signaling Technology (USA).

### Quantification of the RNA level

Total cellular RNA was extracted using a Total RNA Miniprep Purification Kit (GeneMark Biolab, Taiwan) following the manufacturer’s instructions and were transcribed to cDNA with M-MLV reverse transcriptase (Promega, USA). The levels of DENV-2 replication and COX-2 were analyzed by RT-PCR as described previously with the following specific primers: a forward primer, 5′-AAG GTG AGA AGC AAT GC AGC-3′, and a reverse primer, 5′-CCA CTC AGG GAG TTC TCT CT-3′, targeting the DENV-2 NS5 gene^44^. DENV-2 and COX-2 RNA levels were normalized to the cellular glyceraldehyde-3-phosphate dehydrogenase (GAPDH) RNA level of each sample.

### Western blotting

The western blotting procedure was followed from a previous study^45^. The antibodies used in the present study included anti-DENV NS2B (1:3000; GeneTex, Irvine, CA, USA), anti-GAPDH (1:3000; GeneTex, Irvine, CA, USA), anti-COX-2 (1:1000, Cayman, ML, USA), and anti-Myc (1:2000; Abcam, Cambridge, MA, USA). In addition, anti-phosphorylated IκBα, IKKα/β, NF-κB, p38, JNK, and ERK and anti-total IκBα, IKKα, IKKβ, NF-κB, p38, JNK, and ERK antibodies were used (1:1000; Cell Signaling Technology, Inc. Danvers, MA, USA).

### Transfection and luciferase activity assay

To determine the regulation of COX-2 promoter activation during DENV-2 infection, Huh-7 cells were cotransfected with 0.2 μg of pCMV-Renilla-Luc and 1 μg of a series of COX-2 promoter-reporter plasmids, including pCOX-2-Luc COX-2 [COX-2 promoter (WT)], pCOX-2-(ΔNF-κB)-Luc [COX-2 promoter (ΔNF-κB)], and pCOX-2-(ΔNF-κB/C/EBP)-Luc [COX-2 promoter (ΔNF-κB/C/EBP)] for 6 h using T-pro reagent (Ji-Feng Biotechnology CO., Ltd., Taipei, Taiwan) according to the manufacturer’s instructions. To investigate the importance of transcription factor binding regions on the COX-2 promoter during DENV-2 infection, Huh-7 were cotransfected in turn with 0.2 μg of pCMV-Renilla-Luc and 1 μg of pNF-κB-Luc, pC/EBP-Luc, pCRE-Luc, or pAP-1-Luc for 6 h. pCMV-Renilla-Luc served as the internal control for transfection efficiency. To investigate the role of COX-2 in DENV-2 replication, Huh-7 and Huh-7-D2-FLuc-SGR-Neo cells were transfected with different concentrations of the COX-2 expression vector pCMV-COX-2-Myc or shRNA against COX-2, and then, the transfection reagents were exchanged with fresh medium. After 3 days of incubation, cell lysates were subjected to a luciferase activity assay using a Bright-Glo Luciferase Assay system (Promega). To evaluate the activity of DENV-2 NS5 polymerase, Huh-7 cells were cotransfected with pcDNA-NS5-Myc and p(+)RLuc-(−)DV-UTRΔC-Fluc plasmid as described in a previous study^40^. The luciferase activities were determined using a Dual-Glo Luciferase Assay System (Promega).

### PGE_2_ assay

Huh-7 cells were infected with DENV-2 at a multiplicity of infection (MOI) of 0.1, 1, or 5 for 2 h. Supernatant were collected at different times (24, 48, and 72 h). To investigate the effect of NS398 on DENV-2-medicated elevation of PGE_2_ production, Huh-7 cells were infected with DENV-2 at an MOI of 1. Then, the DENV-2-infected cells were treated with NS398 at the indicated concentrations. Supernatants were collected at 72 h. The levels of PGE_2_ were determined using a PGE_2_ high sensitivity ELISA kit (Enzo Life Sciences, USA) according to the manufacturer’s protocol. The absorbance at 405 nm was quantified with a Synergy 2 Multi-Mode microplate reader (BioTek, USA).

### Plaque assay

BHK cells were seeded at a density of 10^5^ cells per well in a 12-well plate and infected with serially diluted virus for 2 h of incubation. The viral inoculum was exchanged with DMEM containing 0.8% methyl-cellulose (Sigma-Aldrich, St. Louis, MO, USA) and incubated for 5 days. The cells were fixed and stained with crystal violet solution (1% crystal violet, 0.64% NaCl, and 2% formalin) for 1.5 h. The crystal violet solution was washed from the cells, and the virus titer was calculated^46^.

### Anti-DENV-2-induced lethality in an ICR suckling mouse model

The strategy for DENV-2-infected ICR suckling mice followed that of previous studies^47^. Briefly, 6-day-old ICR suckling mice were randomly divided into four groups: group 1, intracerebrally injected with 2.5 × 10^5^ pfu of 60 °C heat-inactivated DENV-2 PL046 strain (iDENV); group 2, intracerebrally injected with 2.5 × 10^5^ pfu of DENV-2 and saline (DENV); group 3, intracerebrally injected with 2.5 × 10^5^ pfu of DENV-2 and 1 mg/kg of NS398 (DENV + NS398 1 mg/kg); and group 4, intracerebrally injected with 2.5 × 10^5^ pfu of DENV-2 and 5 mg/kg of NS398 (DENV + NS398 5 mg/kg). Each group comprised 12 suckling mice (n = 12). Mice were injected with NS398 at 1, 3, and 5 dpi. For the 11 day assessment, the DENV-infected mice were injected with NS398 at 1, 3, 5 and 7 dpi. The clinical score, body weight, and survival rate were measured daily after DENV-2 injection. The clinical score was recorded according to the illness symptoms, including 1 for slight loss of weight, 2 for slow motility, 3 for asthenia and anorexia, 4 for lethargy, and 5 for death.

### Statistical analysis

The data are expressed as the mean ± SD. Quantification analysis was performed for three independent experiments, with at least in triplicate samples for each experiment. Groups were compared via Student’s *t-* test. A *P*-value of <0.05 was considered to indicate a statistically significant result.

## Additional Information

**How to cite this article**: Lin, C.-K. *et al*. Cyclooxygenase-2 facilitates dengue virus replication and serves as a potential target for developing antiviral agents. *Sci. Rep.* **7**, 44701; doi: 10.1038/srep44701 (2017).

**Publisher's note:** Springer Nature remains neutral with regard to jurisdictional claims in published maps and institutional affiliations.

## Supplementary Material

Supplemental Information
