# Supplementary material for: Cyclooxygenase‐2 facilitates dengue virus replication and serves as a potential target for developing antiviral agents
Source: Sci Rep. 2017 Mar 20;7:44701. doi: 10.1038/srep44701 (PMC5357798; doi:10.1038/srep44701)
Supplement: Supplemental Information [file srep44701-s1.pdf]

Cyclooxygenase-2 facilitates dengue virus replication and serves as a potential target for developing antiviral agents

Chun-Kuang Lin[1,2], Chin-Kai Tseng[3,4], Yu-Hsuan Wu[3,4], Chih-Chuang Liaw[1,5], Chun-Yu Lin[6,7], Chung-Hao Huang[6,7], Yen-Hsu Chen[6,7,8,9*], Jin-Ching Lee[10,11,12*]

[1] Doctoral Degree Program in Marine Biotechnology, College of Marine Sciences, National Sun Yat-Sen University, Kaohsiung, Taiwan

[2] Doctoral Degree Program in Marine Biotechnology, Academia Sinica, Taipei, Taiwan

[3]Institute of Basic Medical Sciences, College of Medicine, National Cheng Kung University, Tainan, Taiwan

[4]Center of Infectious Disease and Signaling Research, College of Medicine, National Cheng Kung University, Tainan, Taiwan

[5]Department of Marine Biotechnology and Resources, College of Marine Sciences, National Sun Yat-Sen University, Kaohsiung, Taiwan

[6]School of Medicine, College of Medicine, Kaohsiung Medical University, Kaohsiung, Taiwan.

[7]Division of Infectious Diseases, Department of Internal Medicine, Kaohsiung Medical University Hospital, Kaohsiung, Taiwan.

[8]School of Medicine, Graduate Institute of Medicine, Sepsis Research Center, Center for Dengue Fever Control and Research, Kaohsiung Medical University, Kaohsiung, Taiwan.

[9]Department of Biological Science and Technology, College of Biological Science and Technology, National Chiao Tung University, HsinChu, Taiwan.

[10]Department of Biotechnology, College of Life Science, Kaohsiung Medical University, Kaohsiung, Taiwan.

[11]Graduate Institute of Natural Products, College of Pharmacy, Kaohsiung Medical University, Kaohsiung, Taiwan.

[12]Research Center for Natural Products and Drug Development, Kaohsiung Medical University, Kaohsiung, Taiwan.

Running title: Cyclooxygenase-2 induction required for DENV replication

***Corresponding authors:** Jin-Ching Lee and Yen-Hsu Chen

Mail address: Department of Biotechnology, Kaohsiung Medical University, 100, Shih-Chuan 1st Road, San Ming District, 807 Kaohsiung City, Taiwan.

Phone: 886-7-312-1101 ext. 2369 **Fax:** 886-7-312-5339

E-mail: jclee@kmu.edu.tw and infchen@gmail.com

Supplementary material and method

DENV NS2B/NS3 protease assays

To evaluate the effect of PGE_2 on the activity of DENV-2 NS2B/NS3 protease, Huh-7 cells were co-transfected with 0.25 μg of DENV-2 NS2B/NS3 protease reporter vector pEG(Δ4B/5)NLuc and 0.75 μg of pNS2B(G_4SG_4)NS3, a non-autocleavage NS2B/NS3 protease expression vector in the presence of PGE2 at 1, 2.5, and 5 μg/ml. After 3 days treatment, supernatant were collected for measurement of nano luciferase activity using the Nano-Glo® Luciferase Assay system (promega). To normalize the nano luciferase activity of each sample, the transfection mixture contained 0.1 μg of firefly luciferase expression vector (pCMV-FLuc) as a transfection efficiency control.

MTS assay

Huh-7 cells were treated with NS398 at indicated concentrations for 3 days. Cell viability were determine by colorimetric 3-(4,5-dimethylthiazol-2-yl)-5-(3-carboxymethoxyphenyl) -2-(4-sulfophenyl)-2H-tetrazolium (MTS) assay (Promega) according to the manufacturer's instruction (Promega). The plates were re-incubated in 37 °C incubator for 2-4 h, and the absorbance were determined at 490 nm with Synergy 2 Multi-Mode microplate Reader (BioTek, USA).

DENV entry and assembly assay

Huh-7 cells were infected with DENV-2 at MOI of 1 and treated with NS398 at indicated concentatipns for 2 h. The cells were washed by PBS and added complete culture medium for another 46 h. Subsequently, the cellular RNA were subjected to RT-qPCR. To identify the effect of NS398 on DENV-2 assembly, Huh-7 cells were infected with DENV-2 at a MOI of 1, and the DENV-2-infected cells were treated with NS398 at indicated concentrations for 3 days. viral RNA were extracted from supernatant by AllPure Viral DNA/RNA Extraction kit (AllBio Science Incoporated, Taiwan), and the amount of viral RNA were quantified by RT-qPCR.

DENV infection in THP-1 and U937 cells

THP-1 and U937 were pre-treated with phorbol 12-myristate 13-acetate (PMA) for differentiating into macrophages at 30 or 100 nM, respectively. After 3 days, the cells were washed with PBS twice and then infected with DENV-2 at a MOI of 0.5. The DENV-2-infected cells were treated with NS398 at indicated concentrations for 3 days. Cellular RNA were subjected to RT-qPCR for determining relative DENV-2 RNA.

Quantification of positive-sense RNA level

Total cellular RNA was extracted using a Total RNA Miniprep Purification Kit

(GeneMark Biolab, Taiwan) following the manufacturer's instructions. Positive-sense RNA were transcribed to cDNA with M-MLV reverse transcriptase (Promega, USA) using antisense primer. Known amounts of pCMV-NS5-Myc was subjected to RT-qPCR to determine copy numbers. The amounts of intracellular positive-sense DENV-2 RNA were determined by copy number of pCMV-NS5-Myc with cycle number (Ct value) of RT-qPCR.

NS1 ELISA assay

The amount of intracellular NS1 protein level were determined using Platelia Dengue NS1 Ag kit (Bio-Rad, France) following the manufacturer's instructions.

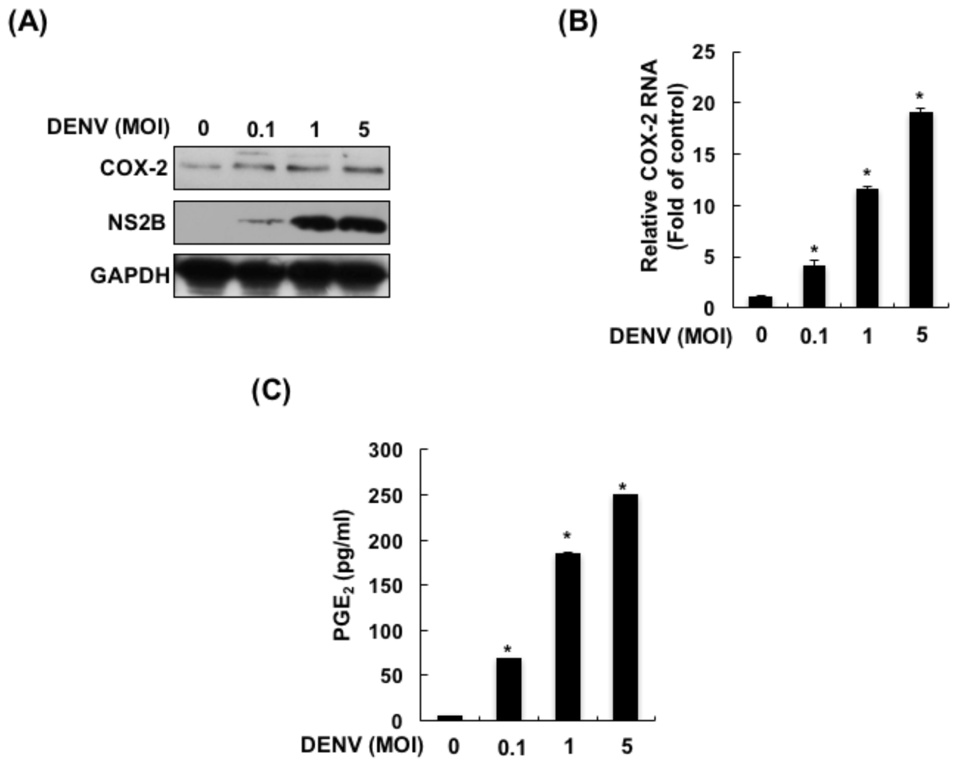

Figure S1. DENV-2 induced COX-2 expression and PGE_2 production in a concentration-dependent manner. DENV-2 infection concentration-dependently induced (A) COX-2 protein expression, (B) RNA level and (C) PGE_2 production. Huh-7 cells were infected with DENV-2 at an MOI of 0.1, 1 and 5. After 3 days, the cell lysates, cellular RNA and supernatants were subjected to Western blotting, RT-qPCR or PGE_2 ELISA, respectively. The relative RNA level of DENV-2 and COX-2 were determine by RT-qPCR following the normalization to cellular *gapdh* mRNA level. All data are indicative of at least three independent experiments, with each measurement performed in triplicate. Error bars are expressed as the mean ± SD of three independent experiments; $^*P < 0.05$.

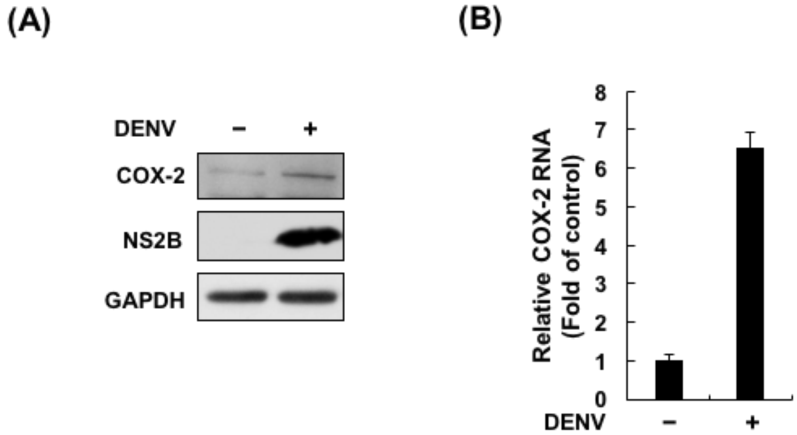

Figure S2. DENV-2 elevated COX-2 expression at an early time point. (A) The COX-2 protein and (B) RNA levels were increased upon DENV-2 infection at 24 hours post-infection (hpi). Huh-7 cells were infected with DENV-2 at an MOI of 5. After 24 hours incubation, cell lysates and cellular RNA were collected and subjected to western blotting or RT-qPCR, respectively. The relative RNA level of COX-2 were determine by RT-qPCR following the normalization to cellular *gapdh* mRNA level. All data are indicative of at least three independent experiments, with each measurement carried out in triplicate. Error bars are expressed as the mean ± SD of three independent experiments; *$P < 0.05$.

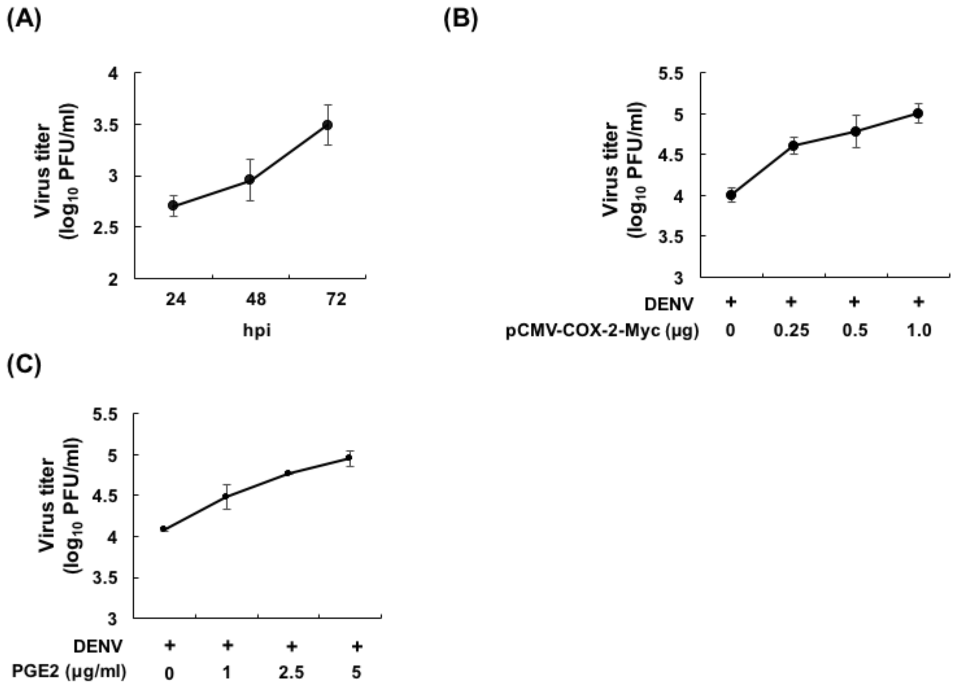

Figure S3. The growth curve of infectious DENV-2, and COX-2 overexpression and PGE$_2$ addition induced DENV-2 propagation at early time point. (A) The growth curve of DENV-2. Huh-7 cells were infected with DENV-2 at an MOI of 1. Supernatants were collected at indicated time point and subjected to a plaque assay. (B and C) COX-2 overexpression and PGE2 addition increased DENV-2 propagation. Huh-7 cells were transfected with pcDNA4/Myc or pcDNA4-COX-2-Myc at the indicated concentrations, and the transfected cells were infected with DENV-2 at an MOI of 1. Huh-7 cells were infected with DENV-2 at an MOI of 1, and the infected cells were treated with PGE$_2$ at the indicated concentrations. After 24 hours incubation, supernatants were collected and subjected to a plaque assay. All data are indicative of at least three independent experiments, with each measurement carried out in triplicate. Error bars are expressed as the mean ± SD of three independent experiments; *$P < 0.05$.

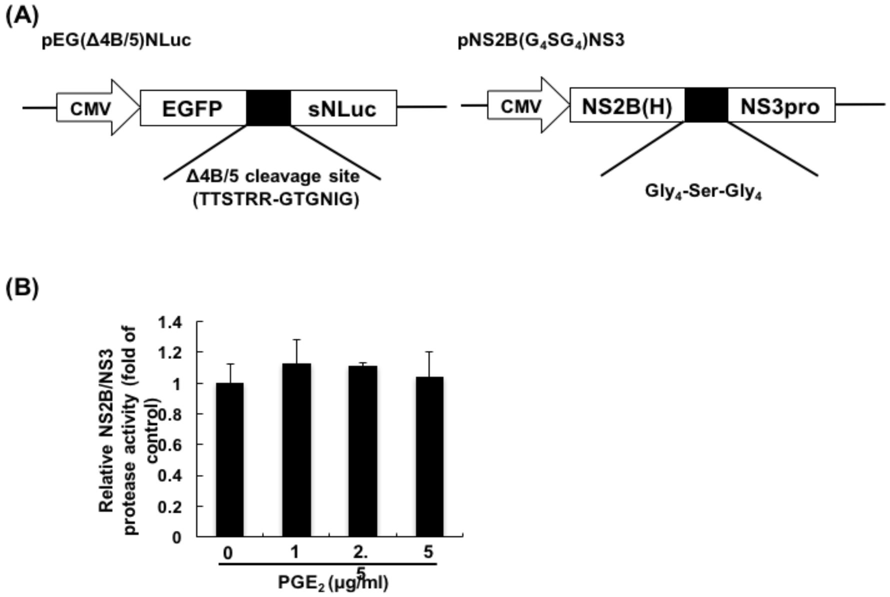

Figure S4. PGE_2 did not affect the DENV-2 protease activity. (A) Schematic diagram of DENV-2 NS2B/NS3 protease activity reporter plasmid, designed as pEG(Δ4B/5)NLuc, and the DENV-2 NS2B/NS3 protease expression vector, designed as pNS2B(G_4SG_4)NS3, without autocleavage activity. NS2B/NS3 protease activity reporter plasmid contains a DENV-2 protease cleavage sequence, which was flanked by *egfp* and *NLuc* genes. (B) PGE_2 did not affect the DENV-2 protease activity. Huh-7 cells were co-transfected with pEG(Δ4B/5)NLuc and pNS2B(G_4SG_4)NS3, followed by treatment of PGE_2 at indicated concentrations for 3 days. The supernatants were subjected to Nano-Glo® Luciferase Assay System. The firefly luciferase expression vector (pCMV-Luc) served as a transfection control for normalization to nano luciferase activity. All data are indicative of at least three independent experiments, with each measurement carried out in triplicate. Error bars are expressed as the mean ± SD of three independent experiments; $*P < 0.05$.

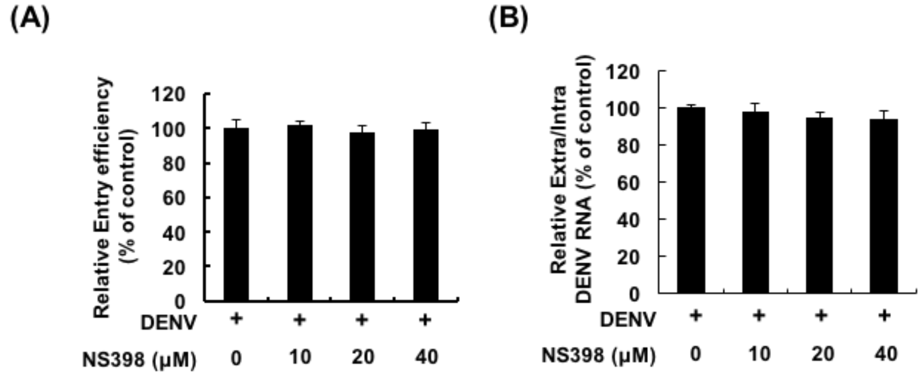

Figure S5. NS398 did not interfere DENV-2 entry and assembly. NS398 did not decrease DENV-2 entry (A) and assembly (B). For viral entry analysis, Huh-7 cells were infected with DENV-2 and treated with NS398 at indicated concentrations for 2 h, and then the culture medium were replaced with complete culture medium following PBS washing. Cellular RNAs were extracted and subjected to RT-qPCR. For viral assembly analysis, Huh-7 cells were first infected with DENV-2 for 2 h, and then the DENV-2-infected cells were treated with NS398 at indicated concentrations for 3 days. Viral RNA in the supernatant and cellular RNA were subjected to RT-qPCR. The effect of NS398 on DENV-2 assembly efficiency was represented through the ratio of the amount of extracellular viral RNA and intracellular viral RNA. All data are indicative of at least three independent experiments, with each measurement performed in triplicate. Error bars are expressed as the mean ± SD of three independent experiments.

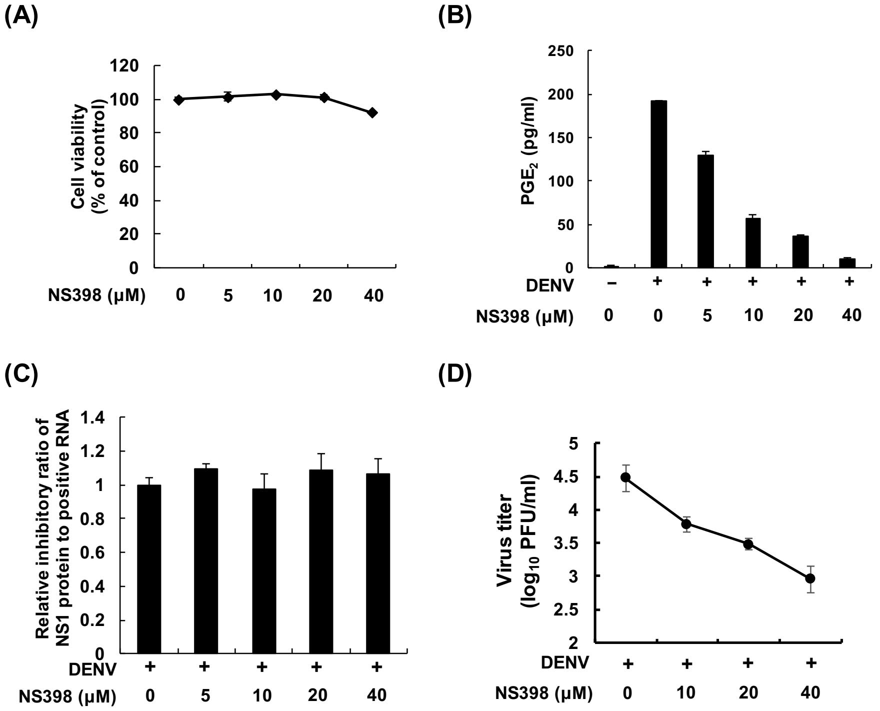

Figure S6. NS398 reduced DENV-2-elevated PGE_2 production without cell cytotoxicity and DENV-2 propagation at early time point. (A) NS398 did not result in significantly cell cytotoxicity. Huh-7 cells were treated with NS398 at indicated concentrations. After 3 days, cell cytotoxicity was analyzed by MTS assay. (B) NS398 decreased DENV-2-mediated elevation of PGE_2 production. Huh-7 cells were infected with DENV-2 at an MOI of 1, and then treated with NS398 at different concentrations (0, 5, 10, 20, and 40 μM) for 3 days. Supernatants were subjected to a PGE_2 ELISA. (C) NS398 reduced DENV RNA synthesis and protein translation in a similar pattern. Huh-7 cells were infected with DENV-2 at an MOI of 1, and the infected cells were treated with NS398 at indicated concentrations for 3 days. The copy numbers of viral positive-sense RNA were determined by RT-qPCR. The NS1 protein level was analyzed

by ELISA. The effect of NS398 on viral protein expression was represented as the relative inhibitory ratio of viral NS1 protein to positive-sense RNA. (D) NS398 suppressed DENV-2 propagation at 24 hpi. Huh-7 cells were infected with DENV-2 at an MOI of 5, and the infected cells were treated with NS398 at indicated concentrations for 24 h. Supernatants were subjected to a plaque assay. All data are indicative of at least three independent experiments, with each measurement performed in triplicate. Error bars are expressed as the mean ± SD of three independent experiments; $*P < 0.05$.

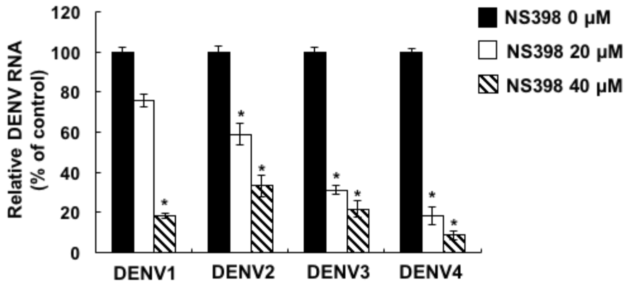

Figure S7. NS398 dose-dependently inhibited 4 serotypes of DENV replication. Huh-7 cells were respectively infected with 4 serotype of DENV at an MOI of 1, and then the cells were treated with different concentrations (0, 20, and 40 μM) of NS398. After 3 days treatment, the levels of 4 serotype of DENV RNA were determined by RT-qPCR. All data are indicative of at least three independent experiments, with each measurement performed in triplicate. Error bars are expressed as the mean ± SD of three independent experiments; $*P < 0.05$.

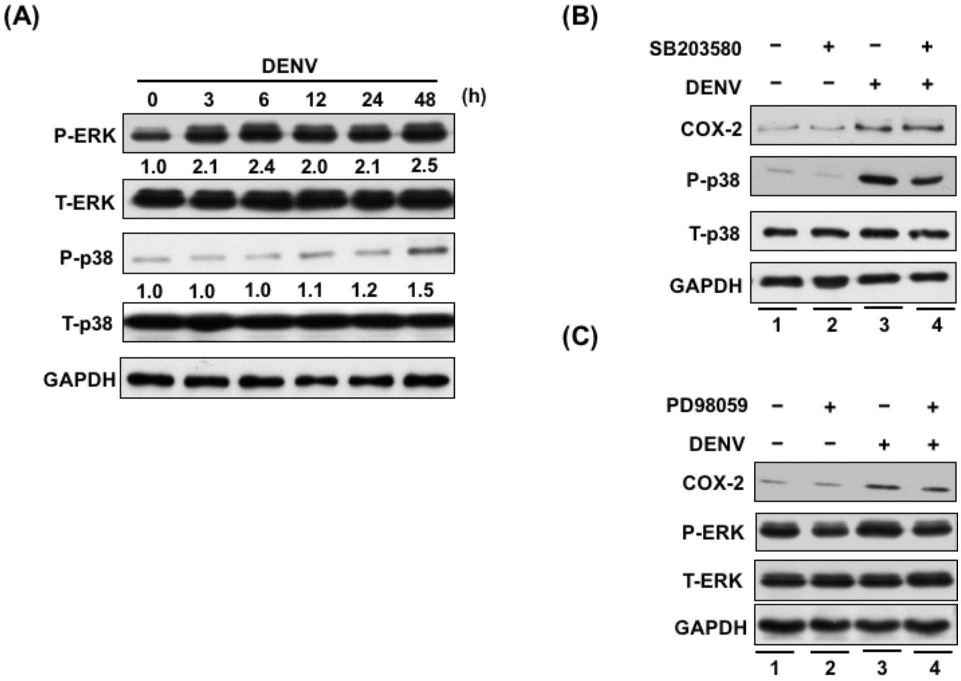

Figure S8. MAPK/ERK and p38 are not responsible for DENV-2-induced COX-2 expression. (A) DENV-2 induced the activation of MAPK/ERK and p38 at 3 and 48 hpi. Huh-7 cells were infected with DENV-2 at an MOI of 1, and the cell lysates were extracted at the indicated time points (0, 3, 6, 12, 24, and 48 h). Western blotting was performed and the relative blot intensities were quantified by densitometry scanning. (B and C) SB203580 and PD98059 did not significantly reduced DENV-2-induced COX-2 expression. Huh-7 cells were pre-treated with DMSO, SB203580 (10 μM), or PD98059 (20 μM) for 2 h, respectively, and then the cells were infected with DENV-2 at an MOI of 1. The cell lysates were extracted after 2 days post-infection (dpi). Western blotting was performed with specific antibodies described in the Material and Method. GAPDH served as an equal loading control. All data are indicative of at least three independent experiments.

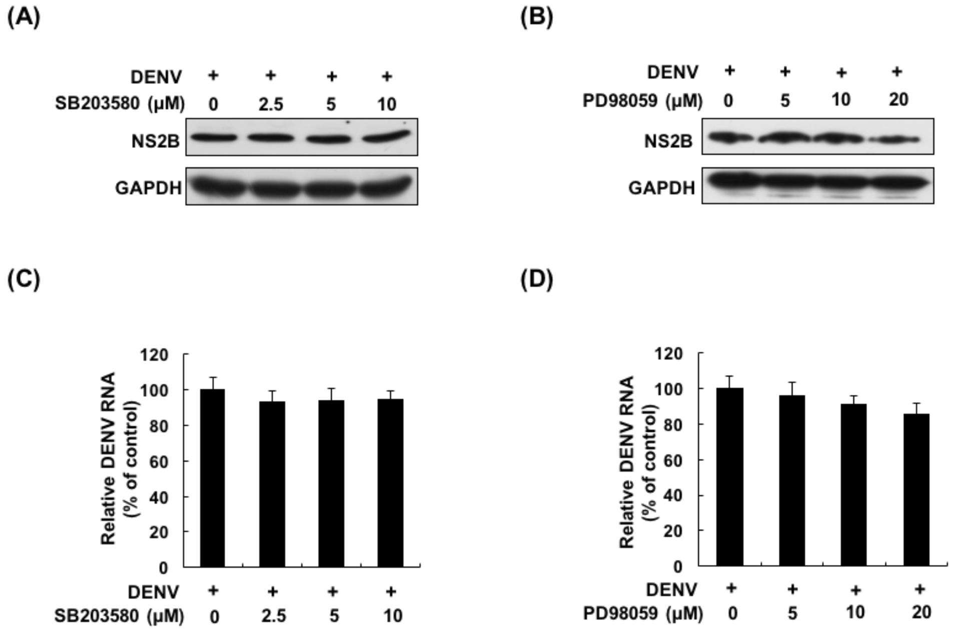

Figure S9. The inhibitors of MAPK/ERK and p38 did not suppress DENV-2 replication. SB203580 and PD98059 did not significantly reduced (A and B) DENV-2 protein synthesis and (C and D) RNA replication. Huh-7 cells were infected with DENV-2 at an MOI of 1, and then the cells were treated with DMSO, SB203580, or PD98059 at indicated concentrations. After 3 days treatment, the cell lysates and cellular RNA were analyzed by western blotting or RT-qPCR, respectively. GAPDH served as an equal loading control. The relative RNA level of DENV-2 was determine by qRT-PCR following the normalization to cellular *gapdh* mRNA level. All data are indicative of at least three independent experiments, with each measurement performed in triplicate. Error bars are expressed as the mean ± SD of three independent experiments.

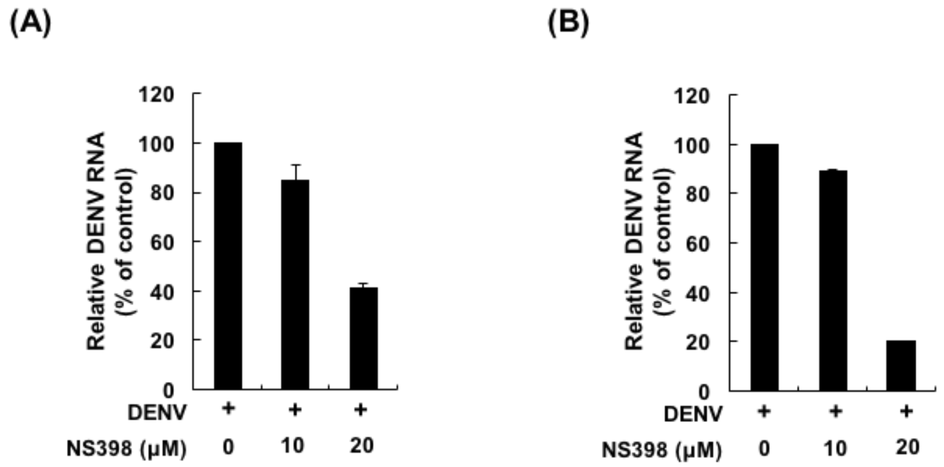

Figure S10. NS398 reduced DENV-2 replication in human monocyte cell line, THP-1 and U937 cells. NS398 decrease DENV-2 replication in (A) THP-1 and (B) U937 cells. PMA-differentiated THP-1 and U937 cells were infected with DENV-2 at a an MOI of 0.5, and then the cells were treated with NS398 at indicated concentrations for 3 days. Cellular RNA were extracted and subjected to RT-qPCR. The relative RNA level of DENV-2 was determine by qRT-PCR following the normalization to cellular *gapdh* mRNA level. All data are indicative of at least three independent experiments, with each measurement performed in triplicate. Error bars are expressed as the mean ± SD of three independent experiments.

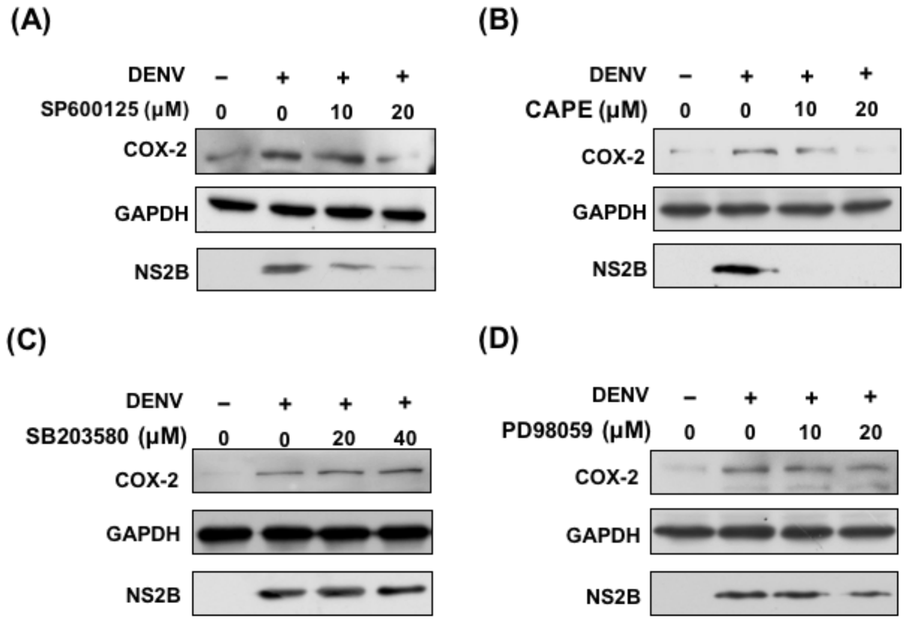

Figure S11. NF-κB and MAPK/JNK are required for DENV-2-induced COX-2 expression and viral replication. (A) SP600125 and (B) CAPE reduced DENV-2-induced COX-2 expression and viral replication. (C) SB203580 and (D) did not affect COX-2 expression and viral replication. Huh-7 cells were infected with DENV-2 at an MOI of 5, and then the cells were treated with specific inhibitors at indicated concentrations for 24 h. Cell lysates were subjected to western blotting with anti-COX-2, anti-NS2B, and anti-GAPDH. GAPDH served as an equal loading control. All data are indicative of at least three independent experiments.
